# Adherence to Pre-Exposure Prophylaxis for HIV Prevention in a Clinical Setting

**DOI:** 10.1371/journal.pone.0157742

**Published:** 2016-06-22

**Authors:** Madeline C. Montgomery, Catherine E. Oldenburg, Amy S. Nunn, Leandro Mena, Peter Anderson, Teri Liegler, Kenneth H. Mayer, Rupa Patel, Alexi Almonte, Philip A. Chan

**Affiliations:** 1 Division of Infectious Diseases, Brown University, Providence, Rhode Island, United States of America; 2 Department of Epidemiology, Harvard School of Public Health, Boston, Massachusetts, United States of America; 3 Department of Behavioral and Social Sciences, Brown University School of Public Health, Providence, Rhode Island, United States of America; 4 Division of Infectious Diseases, University of Mississippi, Jackson, Mississippi, United States of America; 5 Skaggs School of Pharmacy and Pharmaceutical Sciences, University of Colorado, Aurora, Colorado, United States of America; 6 Department of Medicine, University of California San Francisco, San Francisco, California, United States of America; 7 The Fenway Institute, Fenway Health, Boston, MA; Department of Medicine, Beth Israel Deaconess Hospital and Harvard Medical School, Boston, Massachusetts, United States of America; 8 Division of Infectious Diseases, Washington University, St Louis, Missouri, United States of America; Temple University, UNITED STATES

## Abstract

**Background:**

The HIV epidemic in the United States (US) disproportionately affects gay, bisexual, and other men who have sex with men (MSM). Pre-exposure prophylaxis (PrEP) using co-formulated tenofovir disoproxil fumarate (TDF) and emtricitabine (FTC) has demonstrated high efficacy in reducing HIV incidence among MSM. However, low adherence was reported in major efficacy trials and may present a substantial barrier to successful PrEP implementation. Rates of adherence to PrEP in “real-world” clinical settings in the US remain largely unknown.

**Methods:**

We reviewed demographic and clinical data for the first 50 patients to enroll in a clinical PrEP program in Providence, Rhode Island. We analyzed self-reported drug adherence as well as drug concentrations in dried blood spots (DBS) from patients who attended either a three- or six-month follow-up appointment. We further assessed drug concentrations and the resistance profile of a single patient who seroconverted while taking PrEP.

**Results:**

Of the first 50 patients to be prescribed PrEP, 62% attended a follow-up appointment at three months and 38% at six months. Of those who attended an appointment at either time point (70%, n = 35), 92% and 95% reported taking ±4 doses/week at three and six months, respectively. Drug concentrations were performed on a random sample of 20 of the 35 patients who attended a follow-up appointment. TDF levels consistent with ±4 doses/week were found in 90% of these patients. There was a significant correlation between self-reported adherence and drug concentrations (*r* = 0.49, *p* = 0.02). One patient who had been prescribed PrEP seroconverted at his three-month follow-up visit. The patient’s drug concentrations were consistent with daily dosing. Population sequencing and ultrasensitive allele-specific PCR detected the M184V mutation, but no other TDF- or FTC-associated mutations, including those present as minor variants.

**Conclusion:**

In this clinical PrEP program, adherence was high, and self-reported drug adherence accurately reflected drug concentrations as measured by DBS.

## Introduction

The HIV epidemic continues to be a significant public health concern in the United States (US), with over 1.2 million persons currently living with HIV and the number of newly diagnosed cases approaching 50,000 annually [1]. Pre-exposure prophylaxis (PrEP) is a promising approach for preventing HIV among high-risk populations including men who have sex with men (MSM). Clinical trials of emtricitabine (FTC) co-formulated with tenofovir disoproxil fumarate (TDF) as PrEP have demonstrated a greater than 90% reduction of HIV acquisition risk among MSM who were adherent to the medication [2–4]. Adherence has emerged as a critical factor for efficacy, with two major studies in African women demonstrating failure of the intervention to prevent HIV acquisition, due in large part to low adherence [5,6]. Importantly, sub-optimal adherence may also lead to the development of drug resistance [2,5–8] which has the potential to impact subsequent treatment outcomes [9].

Another major finding from the initial efficacy studies was the significant discordance between self-reported adherence and serum drug levels. In the Pre-exposure Prophylaxis Trial for HIV Prevention among African Women (FEM-PrEP) [5] and Vaginal and Oral Interventions to Control the Epidemic (VOICE) [6] studies among African women, self-reported adherence was high (95%), but fewer than 40% had plasma drug concentrations indicative of adherence [5]. The authors attributed low adherence in part to financial and other incentives for clinical trial participation, as many participants in these studies may have enrolled for the benefit of these incentives with little motivation to take or adhere to the study drug [10]. Culturally-specific barriers such as mistrust of the use of experimental drugs may also have affected adherence [11]. Similarly, findings from the iPrEx trial among MSM also indicated a discordance between self-reported adherence and drug concentrations [2]. Consequently, drug concentrations have emerged as the standard for adherence measurement in PrEP studies. Drug concentrations have been successfully measured using serum [5], dried blood spots (DBS) [12], and hair samples [13,14]. In clinical settings, where patients receive no compensation in exchange for taking PrEP, self-report may be a more reliable measure of adherence than research studies have suggested. Data from the US PrEP Demonstration Project showing high adherence, with significant concordance between DBS and self-report, suggest this may be the case [15]. However, participants were screened into the Demonstration Project and provided medications free of charge, a marked difference from clinical implementation settings. Little data are currently available describing adherence in “real-world” PrEP programs, where patients obtain medications through standard clinic procedures, and accuracy of self-report as a measure of adherence.

In 2013, we implemented a clinical PrEP program in Providence, Rhode Island. We evaluated FTC and TDF concentrations by DBS and compared to self-reported adherence among patients. We also describe drug concentrations in a single patient who seroconverted while on PrEP, as well as the resistance profiles generated by commercial genotyping and minor variant assays. This is one of the first reports of adherence, using drug concentration levels, in which data were collected during routine clinical care.

## Materials and Methods

We reviewed medical records of the first 50 patients receiving PrEP care at an outpatient infectious diseases clinic in Providence, Rhode Island, between February 2013 and June 2014. Patients provided written informed consent to participate in this study. Consent procedure and study protocol were approved by The Miriam Hospital Institutional Review Board. Data collected included age, race and ethnicity, insurance status, referral source, indications for PrEP, attendance at follow-up appointments, HIV serostatus, and self-reported adherence. HIV serostatus was determined by third-generation antibody testing (ADVIA Centaur HIV 1/O/2, Siemens Medical Solutions USA, Inc., Malvern, PA). Patients were scheduled for follow-up appointments every three months, in accordance with guidelines from the Centers for Disease Control and Prevention (CDC) for administering PrEP [16]. Medical providers assessed self-reported adherence at each visit by verbally asking patients the number of doses missed in the past seven and 30 days. Recall over seven- and 30-day periods is commonly used as a measure of adherence, and these measures have demonstrated reliability when compared to objective measures of adherence [17].

**Fig 1:**
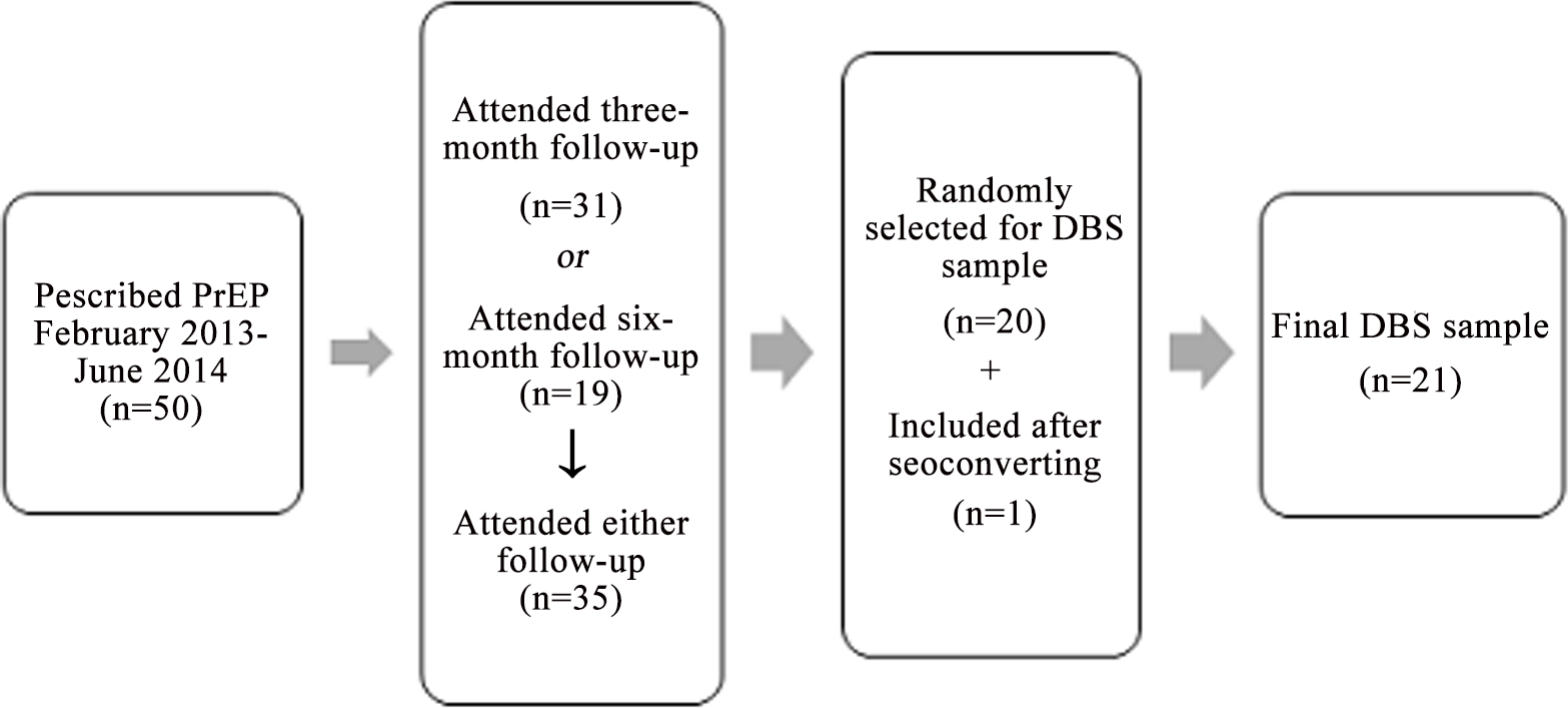
Sample selection for dried blood spot (DBS) analysis.

Thirty-five of the first 50 PrEP patients attended three- or six-month follow-up appointments; of these, 20 patients were randomly selected for DBS samples to measure drug concentrations. One additional patient was included in the DBS study after seroconverting while taking PrEP (**Fig 1**). Blood for DBS analysis was drawn during the course of routine clinical venipuncture; DBS analysis was conducted as described elsewhere [18]. In red blood cells (as measured by DBS), TDF and FTC exist in the phosphorylated forms tenofovir-diphosphate (TFV-DP) and emtricitabine-triphosphate (FTC-TP) [19]. Unlike free drug in plasma, measurement of the intracellular forms correlates with long-term, rather than recent, drug exposure, representing drug adherence over a longer time period [18]. In the present study, we limit the focus of our analyses to TFV-DP levels, which measure cumulative adherence over the preceding one to two months. In contrast, FTC-TP levels only reflect dosing within the past 48 hours. A TFV-DP concentration of ≥700fmol/punch, corresponding to an average of four or more doses per week, is associated with a 96% reduction in HIV transmission risk (95% confidence interval [CI]: 86, 100) [12].

To compare patients who were and were not retained in care, and did and did not have DBS samples, we performed two-tailed t-tests for continuous variables and Fisher’s exact test for categorical variables across demographic and behavioral categories. Pearson’s correlation coefficient was calculated to measure the correlation between self-reported adherence and drug concentrations. Significance was determined for all tests at a two-tailed *p*-value of <0.05. Statistical analyses were conducted using Stata/SE 13.1 (StataCorp LP, College Station, TX).

For the patient who seroconverted, commercial genotyping was performed using the Celera Diagnostics ViroSeq HIV-1 Genotyping System (Applied Biosystems, Foster City, CA). Detection and quantification of drug resistance mutations M184V, K70E and K65R when present as minor variants was performed using allele-specific PCR as described elsewhere [20].

## Results

Of the first 50 patients prescribed PrEP, 58% were non-Hispanic white, 26% were non-Hispanic black or African American, 26% were Hispanic or Latino, and 8% were another race. Eighty-eight percent were male, the majority (95%) of whom were MSM. The average age of the first 50 patients was 33.8 years (range: 18–58). All but one of the first 50 patients were insured. The mean annual income was $49,190, with a range of $0 to $350,000. Patients were referred to the PrEP program by the publicly-funded STD clinic (40%), the onsite HIV outpatient center (26%), outpatient physicians (20%), or other referral sources (14%), which included friends, community organizations, and the onsite post-exposure prophylaxis (PEP) program. A total of 88% (n = 38) and 82% (n = 31) of the first 50 patients reported taking PrEP at three and six months, respectively. Reasons for discontinuing PrEP included side effects (n = 3), moving (n = 3), and a reduction in behaviors associated with HIV acquisition (n = 1). A total of 70% (n = 35) of patients who were initially prescribed PrEP successfully attended a follow-up visit at either three or six months. Those who were retained in care three or six months after PrEP initiation did not differ significantly from those not retained in care on any measured demographic variables.

Thirty-one patients attended a three-month follow-up appointment and 19 attended a six-month follow-up appointment. Among those who attended visits at three or six months (n = 35), the mean self-reported drug adherence across follow-up visits was 6.2 and 26.8 doses taken in the previous seven and 30 days, respectively. Those included in the DBS sample (n = 21) did not differ significantly from those who were not included on the basis of age, race and ethnicity, gender, indications for PrEP, referral source, attendance of follow-up appointments, or self-reported drug adherence in the past seven days (**Table 1**). Patients in the DBS sample had higher mean self-reported drug adherence in the past 30 days (28.5 doses taken) compared to patients not included in the DBS sample (24.0 doses taken; *p* = 0.03).

**Table 1:** Drug adherence and characteristics of patients with and without dried blood spots (DBS) who were retained in PrEP care at three and six months.

	No DBS	DBS	Total	P-value
	n = 14 ^a^	n = 21^b^	n = 35	
	n (%)			
**Race/ethnicity**				
White, non-Hispanic	10 (64.3)	13 (61.9)	22 (62.9)	1.00
Black, non-Hispanic	1 (7.14)	1 (4.76)	2 (5.7)	
Hispanic/Latino	3 (21.4)	5 (23.8)	8 (22.9)	
Other	1 (7.14)	2 (9.52)	3 (8.6)	
**Gender**				
Male	13 (92.9)	20 (95.2)	33 (94.3)	1.00
**Indications for PrEP**^c^				
MSM	13 (92.9)	19 (90.5)	32 (91.4)	1.00
MSMW	0	1 (4.8)	1 (2.9)	
WSM	1 (7.1)	1 (4.8)	2 (5.7)	
Serodiscordant couple^d^	7 (50.0)	9 (42.9)	16 (45.7)	0.74
**Insurance status**				
No insurance	0	1 (4.8)	1 (2.9)	0.49
Blue Cross Blue Shield	8 (57.1)	8 (38.1)	16 (45.7)	
United Healthcare	3 (21.4)	2 (9.52)	5 (14.3)	
Medicare/Medicaid	0	2 (9.52)	2 (5.7)	
Other	3 (21.4)	8 (38.1)	11 (31.4)	
**Referral source**				
STD Clinic	5 (35.7)	7 (33.3)	14 (37.8)	0.43
Immunology Center	6 (42.9)	5 (23.8)	11 (29.7)	
Outpatient physician	2 (14.3)	3 (14.3)	3 (13.5)	
Other	1 (7.1)	6 (28.6)	7 (18.9)	
**Seronegative at time of DBS**	14 (100)	20 (95.2)	34 (97.14)	0.60
**Attended three-month follow-up**	11 (78.6)	20 (95.2)	31 (88.6)	0.17
**Attended six-month follow-up**	9 (64.3)	10 (47.6)	19 (54.29)	0.27
**Attended three- or six-month follow-up**	6 (42.9)	9 (42.9)	15 (42.9)	1.00
	***Mean (range)***			
**Age at PrEP initiation, years**	36.0 (18–58)	33.9 (21–57)	34.9 (18–58)	0.53
**Annual income (in $1,000)**	52.0 (0–110)	52.0 (0–350)	52.0 (0–350)	1.00
**Self-reported doses taken, past 7 days**	5.59 (0–7)	6.52 (5–7)	6.16 (0–7)	0.07
**Self-reported doses taken, past 30 days**	24.0 (0–30)	28.5 (24.6–30)	26.8 (0–30)	**0.03**
**Initiation to DBS, months**	—	4.4 (1.78–13.3)	—	
**TFV-DP, fmol/punch**	—	1493.5 (31.9–4141.1)	—	
**FTC-TP, pmol/punch** (n = 19)	—	0.296 (0.190–0.466)	—	

–Measure does not apply to this group; Note: **bold values** indicate significance at two-tailed *p* = 0.05 level

a–Group excluded from DBS sample n = 14 for all measures except self-reported adherence in past 7 and 30 days (n = 13) due to missing data for one patient

b–DBS sample n = 21 for all measures except FTC-TP concentration (n = 19) as a result of two blood levels containing below the detectable level of FTC-TP

c–MSM—man who has sex with men, MSMW—man who has sex with men and women, WSM—woman who has sex with men

d–Belonging to a serodiscordant couple is not mutually exclusive with other sexual behavior categories

**Fig 2:**
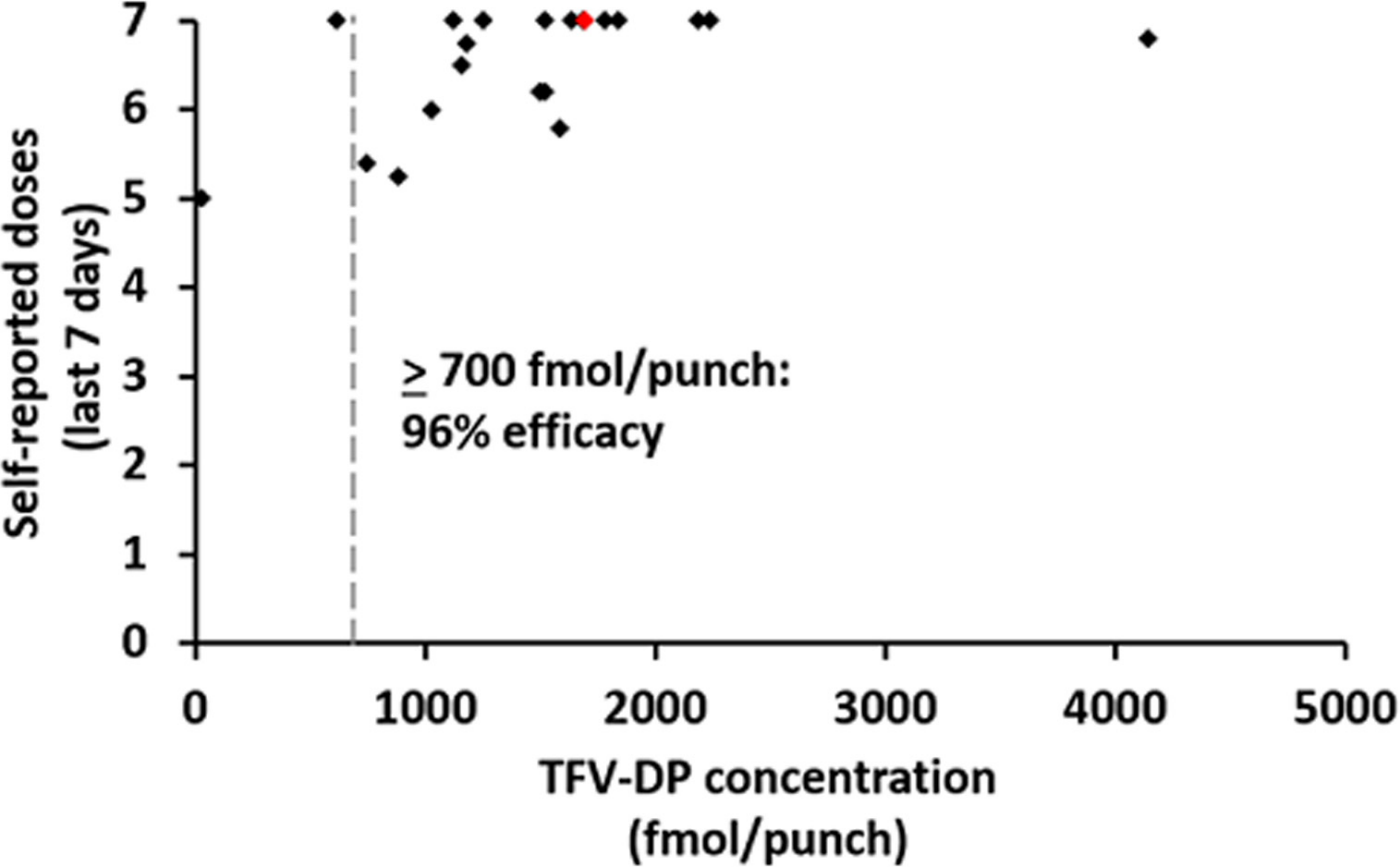
Tenofovir-diphosphate (TFV-DP) concentration and self-reported number of doses taken in the past seven days among pre-exposure prophylaxis (PrEP) patients (n = 21). Red marker indicates the patient who seroconverted while taking PrEP.

DBS samples were collected an average of 4.4 months (range: 1.8–13.3) after PrEP initiation. Based on DBS analyses, patients had a mean TFV-DP concentration of 1493 fmol/punch (range: 31.9–4141.1 fmol/punch) and a mean FTC-TP concentration of 0.296 pmol/punch (range: 0.190–0.466 pmol/punch). FTC-TP concentrations for two patients fell below the level of quantification (BLQ; 0.100 pmol/punch); given that no measurements were obtained, these two patients were not included in calculating the mean. TFV-DP concentrations for the two patients with BLQ FTC-TP were 31.9 and 619.1fmol/punch. By TFV-DP concentration, 5% (1/21) of patients took fewer than two doses per week (BLQ-349 fmol/punch), 5% (1/21) of patients took two to three doses per week (350–699 fmol/punch), and 90% (19/21) of patients took four or more doses per week (±700 fmol/punch). TFV-DP concentration and self-reported adherence in the past seven days were significantly correlated (*r* = 0.49, *p* = 0.02; **Fig 2**); however, no significant correlation emerged between TFV-DP concentration and past-30 day adherence (*r* = -0.13, *p* = 0.58).

Of the initial 50 patients prescribed PrEP, one was found to be HIV-positive by antibody testing at the three-month follow-up appointment. A third-generation HIV antibody test was nonreactive on the day PrEP was initiated. The patient had no signs or symptoms of acute HIV infection and did not have a HIV RNA test performed. A DBS sample taken at the time of HIV diagnosis, three months after PrEP initiation, revealed a concentration of 1696.4 fmol/punch TFV-DP, consistent with daily dosing. The patient tolerated the medication well and reported no missed doses. Prior to initiating PrEP and while taking PrEP, the patient reported engaging in condomless anal sex with multiple partners. An HIV antibody test conducted at the patient’s three-month follow-up visit was reactive, and subsequent viral load testing indicated a viral load of 242 copies/mL while on PrEP. A commercial genotype analysis was performed and successfully sequenced, despite the low viral load. The following mutations associated with nucleoside reverse transcriptase inhibitor (NRTI) drug resistance were detected: D67N, M184V, T215S, and K219Q. The M184V mutation was also detected by AS-PCR, and the protease-associated mutation L10I was also noted.

## Discussion

The present study is among the first to measure PrEP adherence by drug concentrations among early adopters in a clinical program and outside the controlled environment of a clinical trial in the US. Self-reported adherence was high and significantly correlated with drug concentrations obtained via DBS testing. PrEP adherence is critical for optimal intervention efficacy. In efficacy trials, the prevalence of seroconversions ranged from less than 1% in the immediate PrEP arm of the PROUD study to 6% in the oral TDF/FTC arm of the VOICE study, paralleling respective high and low adherence in these trials [4,6]. Pharmacokinetic studies indicate decreasing efficacy of TDF/FTC as PrEP with less than daily dosing; among MSM, taking four doses per week is associated with 96% efficacy, while taking two doses per week is associated with 76% efficacy [19]. In the iPrEx open-label extension (OLE) study, no seroconversions were recorded when TFV-DP concentration in DBS was above 700 fmol/punch, indicative of at least four doses per week, and only one seroconversion occurred at 350 fmol/punch TFV-DP, indicative of two to three doses taken per week [12]. According to these data, PrEP should successfully, with 96% efficacy, prevent HIV in the majority (90%) of the patients in our clinical cohort. Furthermore, results from the iPrEx OLE study indicated that drug DBS concentration four weeks after initiation was significantly associated with sustained adherence over time [12], suggesting that adherence levels in this clinical sample, taken an average of 4.8 months after initiation, are likely to be consistent with both earlier and future adherence.

Demonstration projects and open label studies have provided some insight into PrEP adherence in clinical settings. In the iPrEx OLE, TFV-DP concentrations consistent with taking no doses, fewer than two doses, two to three doses, and four to six, and seven doses per week were detected at 25%, 26%, 12%, 21%, and 12% of study visits, respectively [12]. The US PrEP Demonstration Project noted high adherence based on DBS analysis, reporting 98% of participants with detectable TFV-DP concentration, and TFV-DP concentration consistent with four or more doses per week in 80–86% of participants across follow-up time [15,21]. However, participants were screened into these demonstration studies on the basis of interest and medication was provided at no cost. The results of the current study in an exclusively clinical PrEP program suggest that adherence levels are high in individuals retained in care.

Early results of the PrEP efficacy trials suggested that adherence would be a major obstacle during implementation [5,6]. However, our findings suggest that patients in real-world clinical settings who are retained in care are highly adherent. One possible reason for this difference is that participants in research studies are financially or otherwise compensated for their participation, which may serve as an incentive to attend study appointments, regardless of motivation for drug adherence. Lower adherence in clinical trials may also be affected by the uncertainty of study drug efficacy and the possibility of receiving a placebo treatment [10]. In the clinical setting, patients who are non-adherent are unlikely to be seeking prescription refills and may not be motivated to attend follow-up appointments. As a result, retention in care becomes a reliable indicator of adherence. Our data suggest that retention in care, in conjunction with medication adherence, is a key component of successful PrEP implementation and should be a major focus of future intervention efforts.

Suboptimal adherence to TDF/FTC for PrEP has contributed to reduced efficacy and, less commonly, the emergence of PrEP-selected mutations for drug resistance. Most drug resistance mutations in other PrEP studies were observed among those determined to have had acute HIV infection at study entry [8,20]. Consequently, baseline infection appears to be a greater factor in generating resistance than infection acquired during PrEP treatment. Nonetheless, low drug concentrations may facilitate development of mutations associated with FTC or TDF resistance. Of the 305 total PrEP seroconversion cases documented in studies published through July 2015, 6% demonstrated HIV resistance mutations [22]. All studies reporting resistance mutations in this time period identified the reverse transcriptase M184V/I mutation. However, recent evidence suggests that drug resistance mutations associated with use of TDF/FTC as PrEP decay rapidly; among seroconverters in the intervention arms of the Partners PrEP, Fem-PrEP, and iPrEx studies, PrEP-selected mutations failed to persist for longer than six months after ceasing PrEP treatment [20,23,24].

The current study presents a single case of seroconversion during PrEP clinical care. It is unclear whether the patient had acute HIV infection at baseline or seroconverted while on PrEP. Initiation of TDF/FTC as PrEP during acute HIV infection may facilitate development of drug resistance [7,16,20]. The patient had several mutations associated with drug resistance; however, only the M184V mutation was associated with FTC resistance. The other mutations were thymidine analog mutations (TAMs) associated with NRTIs other than tenofovir. The most likely cause of these mutations is transmitted drug resistance, rather than drug resistance acquired while on PrEP. Transmitted drug resistance, especially multi-class drug resistance, may decrease the efficacy of PrEP for preventing HIV acquisition [25]. This case is among the first to demonstrate high PrEP adherence in a seroconverter in clinical practice, highlighting the potential difficulty in determining the source of drug resistance in cases of seroconversion during PrEP treatment.

The seroconversion case underscores the importance of excluding acute HIV infection prior to PrEP initiation. Current CDC guidelines for PrEP recommend screening for acute HIV infection, which may include clinical screening for signs and symptoms or viral load testing [16]. At our clinic site, viral load testing is only performed for patients who report signs or symptoms consistent with acute HIV infection. We have found that insurance carriers may not cover viral load testing in patients without symptoms, despite the fact that up to 25% of individuals in the acute stage of infection report no signs and symptoms [26]. The case presented here suggests that a higher degree of clinical suspicion for acute HIV infection may be needed in certain patients to avoid initiating PrEP during acute infection. Adoption of fourth generation HIV antigen/antibody testing may improve detection of acute HIV infection among prospective PrEP patients. Notably, the patient’s viral load was low, indicative of treatment with TDF/FTC, which likely had the added benefit of preventing HIV transmission to others [27]. The patient attained viral load suppression within eight months after initiating a darunavir-boosted regimen with TDF/FTC.

A limitation of this study is the small sample size. Future studies should evaluate PrEP adherence among a larger cohort and among different patient populations. The study site was an infectious disease clinic; the relationship between retention in care and adherence may differ in other settings (e.g., primary care clinics) where patients receive other healthcare services in addition to PrEP. Further, the patient sample in this study was primarily non-Hispanic white or Hispanic; adherence, retention in care, and other outcomes of PrEP treatment may vary among other racial and ethnic groups.

This study is among the first to demonstrate a strong correlation between PrEP self-reported adherence and drug concentrations in an exclusively clinical setting. Contrary to low adherence reported in early clinical trials, we report high adherence to PrEP among a sample of patients retained in care. PrEP patients who are successfully retained in care and who attend follow-up appointments are likely to be adherent to this treatment. Future research and interventions should consider retention in PrEP care as a target for improvement.
